# Supplementary material for: An updated antennal lobe atlas for the yellow fever mosquito Aedes aegypti
Source: PLoS Negl Trop Dis. 2020 Oct 20;14(10):e0008729. doi: 10.1371/journal.pntd.0008729 (PMC7575095; doi:10.1371/journal.pntd.0008729)
Supplement: S1 Table — (DOCX) [file pntd.0008729.s002.docx]

**S1 Table. Revised names for all glomeruli annotated in the male antennal lobe model of Ignell *et al*. (2005)**

| **Male Antennal Lobe** | |
| --- | --- |
| **Glomerulus name in** | **Glomerulus** |
| **Ignell *et al*. (2005)** | **name in this atlas** |
| V1 | AD3 |
| V2 | AD1 |
| V3 | AD2 |
| AL1 | AL1 |
| AL2 | AL2 |
| AL3 | AL3 |
| AL4 * | AL4 * |
| AM1 | AM1 |
| AM2 | AM2 |
| AM3 | AM3 |
| AM4 * | AC2 * |
| AM5 | AC3 |
| AM6 | D1 |
| AM7 * | D3 * |
| AD1 | D4 |
| AD2 | D5 |
| AD3 | D2 |
| AD4 | - |
| AD5 | PM1 |
| AD6 | PM2 |
| AD7 | PM3 |
| AC1 | PC1 |
| AC2 | AC1 |
| AC3 * | AD5 * |
| AC4 * | AD4 * |
| PM1 * | AC4 * |
| PM2 | PV1 |
| PM3 * | PM6 * |
| PM4 * | PM5 * |
| PC1 | PC4 |
| PC2 | CD4 |
| PC3 | CD5 |
| MD1 | MD1 |
| MD2 | MD3 |
| MD3 * | MD4 * |
| PD1 | PM4 |
| PD2 | V1 |
| PD3 | V2 |
| PD4 | V4 |
| PD5 | V3 |
| PD6 | V6 |
| PD7 | V7 |
| PD8 | V8 |
| PL1 | PL2 |
| PL2 | PL1 |
| LC1 | PL3 |
| CD1 | CD1 |
| CD2 | CD3 |
| CD3 | CD2 |

* Spatially variant

(-) Unable to definitively assign a revised name for this glomerulus due its ambiguous position between glomerular spatial groups
