# Supplementary material for: An updated antennal lobe atlas for the yellow fever mosquito Aedes aegypti
Source: PLoS Negl Trop Dis. 2020 Oct 20;14(10):e0008729. doi: 10.1371/journal.pntd.0008729 (PMC7575095; doi:10.1371/journal.pntd.0008729)
Supplement: S2 Table — (DOCX) [file pntd.0008729.s003.docx]

**S2 Table.** Quantitative comparison of glomerular volume in the female and male antennal lobe.

| **Glomerulus** | **Female** | | ***n*** | **Male** | | ***n*** | ***U*-test for right**  **and left AL of female** | ***U*-test for right**  **and left AL of male** | ***U*-test for female**  **and male** |
| --- | --- | --- | --- | --- | --- | --- | --- | --- | --- |
|  | **Volume**  **(10^3^ μm^3^)** | **Relative volume**  **(%)** |  | **Volume**  **(10^3^ μm^3^)** | **Relative volume**  **(%)** |  | ***P*-value** | ***P*-value** | ***P*-value** |
| AC1 | 1.34 ± 0.46 | 1.07 ± 0.31 | 10 | 1.24 ± 0.52 | 1.48 ± 0.66 | 9 | 0.310 | 1.000 | 0.133 |
| AC3 | 1.24 ± 0.47 | 0.99 ± 0.35 | 10 | 1.05 ± 1.04 | 1.20 ± 1.17 | 8 | 0.841 | 0.486 | 0.460 |
| AL1 | 3.14 ± 1.03 | 2.56 ± 0.83 | 10 | 2.04 ± 1.15 | 2.38 ± 1.31 | 10 | 0.421 | 0.421 | 0.529 |
| AL2 | 2.04 ± 0.73 | 1.66 ± 0.58 | 10 | 1.19 ± 0.72 | 1.38 ± 0.86 | 10 | 0.421 | 1.000 | 0.280 |
| AL3 | 1.73 ± 0.74 | 1.37 ± 0.54 | 10 | 0.78 ± 0.54 | 0.88 ± 0.55 | 9 | 0.841 | 0.730 | 0.065 |
| AM1 | 3.89 ± 1.31 | 3.10 ± 0.78 | 10 | 1.86 ± 1.00 | 2.19 ± 1.24 | 10 | 0.151 | 0.691 | 0.089 |
| AM2 | 2.15 ± 1.07 | 1.73 ± 0.86 | 10 | 1.95 ± 0.75 | 2.29 ± 0.85 | 10 | 0.691 | 0.222 | 0.105 |
| AM3 | 1.31 ± 0.61 | 1.06 ± 0.47 | 10 | 1.43 ± 0.36 | 1.67 ± 0.36 | 10 | 1.000 | 0.421 | 0.004* |
| AD1 | 4.71 ± 0.83 | 3.82 ± 0.56 | 10 | 2.46 ± 0.67 | 2.89 ± 0.76 | 10 | 0.421 | 0.056 | 0.005* |
| AD2 | 1.33 ± 0.68 | 1.09 ± 0.58 | 10 | 0.81 ± 0.64 | 0.93 ± 0.67 | 10 | 1.000 | 0.548 | 0.280 |
| AD3 | 1.03 ± 0.58 | 0.84 ± 0.46 | 10 | 0.76 ± 0.55 | 0.90 ± 0.68 | 9 | 0.222 | 0.111 | 1.000 |
| VC1 | 4.18 ± 1.72 | 3.33 ± 1.30 | 10 | 1.82 ± 0.53 | 2.14 ± 0.64 | 10 | 1.000 | 0.310 | 0.036* |
| VC2 | 2.44 ± 0.98 | 1.95 ± 0.72 | 10 | 1.51 ± 0.68 | 1.75 ± 0.79 | 10 | 0.691 | 0.151 | 0.029 |
| VC3 | 3.39 ± 2.26 | 2.64 ± 1.57 | 10 | 1.31 ± 0.67 | 1.50 ± 0.64 | 10 | 0.548 | 0.691 | 0.123 |
| VC4 | 2.53 ± 2.58 | 1.99 ± 2.05 | 10 | 1.27 ± 0.62 | 1.50 ± 0.76 | 10 | 0.841 | 0.841 | 0.912 |
| VC5 | 2.38 ± 1.55 | 1.88 ± 1.03 | 10 | 1.60 ± 0.62 | 1.87 ± 0.72 | 10 | 0.008* | 0.841 | 0.436 |
| VC6 | 3.13 ± 2.15 | 2.52 ± 1.60 | 10 | 2.77 ± 1.15 | 3.23 ± 1.29 | 10 | 0.841 | 1.000 | 0.315 |
| VC7 | 1.03 ± 0.85 | 0.84 ± 0.67 | 10 | 0.87 ± 0.54 | 1.02 ± 0.61 | 9 | 0.032* | 0.016* | 0.315 |
| VC8 | 1.07 ± 0.69 | 0.87 ± 0.53 | 9 | 0.51 ± 0.26 | 0.60 ± 0.30 | 10 | 0.730 | 0.151 | 0.356 |
| VC9 | 0.73 ± 0.48 | 0.61 ± 0.43 | 9 | 0.47 ± 0.27 | 0.55 ± 0.31 | 9 | 0.730 | 0.191 | 0.730 |
| PC1 | 0.55 ± 0.41 | 0.46 ± 0.34 | 10 | 0.40 ± 0.32 | 0.47 ± 0.38 | 10 | 0.032 | 0.421 | 0.912 |
| PC2 | 0.66 ± 0.37 | 0.55 ± 0.30 | 9 | 0.68 ± 0.60 | 0.78 ± 0.68 | 10 | 0.064 | 0.691 | 0.720 |
| PC3 | 0.65 ± 0.36 | 0.53 ± 0.31 | 8 | 0.40 ± 0.20 | 0.46 ± 0.23 | 9 | 0.886 | 0.905 | 0.673 |
| PC4 | 0.64 ± 0.41 | 0.53 ± 0.37 | 10 | 0.48 ± 0.26 | 0.55 ± 0.28 | 9 | 0.056 | 0.111 | 0.661 |
| PV1 | 2.30 ± 0.71 | 1.86 ± 0.51 | 10 | 1.98 ± 0.59 | 2.35 ± 0.76 | 10 | 0.548 | 0.056 | 0.166 |
| PV2 | 2.01 ± 1.21 | 1.61 ± 0.90 | 10 | 1.91 ± 1.44 | 2.20 ± 1.60 | 10 | 0.056 | 1.000 | 0.481 |
| PV3 | 2.24 ± 1.43 | 1.80 ± 1.12 | 10 | 1.81 ± 1.25 | 2.07 ± 1.41 | 10 | 0.841 | 0.151 | 0.912 |
| PV4 | 0.98 ± 0.51 | 0.79 ± 0.40 | 10 | 0.68 ± 0.38 | 0.79 ± 0.43 | 10 | 0.151 | 0.310 | 0.796 |
| PV5 | 0.75 ± 0.62 | 0.59 ± 0.44 | 10 | 0.61 ± 0.25 | 0.71 ± 0.27 | 10 | 0.841 | 0.691 | 0.248 |
| PM1 | 0.64 ± 0.44 | 0.53 ± 0.36 | 10 | 0.60 ± 0.27 | 0.69 ± 0.30 | 9 | 0.222 | 0.191 | 0.243 |
| PM2 | 0.74 ± 0.45 | 0.61 ± 0.37 | 9 | 0.59 ± 0.27 | 0.68 ± 0.35 | 9 | 0.413 | 0.730 | 0.667 |
| PM3 | 0.88 ± 0.53 | 0.73 ± 0.45 | 9 | 0.53 ± 0.44 | 0.63 ± 0.57 | 10 | 0.413 | 0.222 | 0.400 |
| PM4 | 0.92 ± 0.46 | 0.77 ± 0.42 | 9 | 0.49 ± 0.30 | 0.58 ± 0.35 | 9 | 0.556 | 1.000 | 0.297 |
| PL1 | 2.14 ± 1.32 | 1.77 ± 1.09 | 10 | 1.61 ± 1.04 | 1.85 ± 1.10 | 10 | 1.000 | 0.056 | 0.971 |
| PL2 | 1.70 ± 1.07 | 1.40 ± 0.87 | 10 | 1.75 ± 1.29 | 2.05 ± 1.54 | 10 | 0.008* | 0.095 | 0.481 |
| PL3 | 2.95 ± 1.26 | 2.44 ± 1.07 | 10 | 1.70 ± 0.86 | 2.04 ± 1.12 | 10 | 1.000 | 0.548 | 0.393 |
| PL4 | 2.10 ± 1.12 | 1.74 ± 0.92 | 10 | 1.22 ± 1.16 | 1.46 ± 1.41 | 10 | 0.421 | 0.421 | 0.248 |
| PL5 | 1.21 ± 0.87 | 0.97 ± 0.62 | 10 | 1.06 ± 0.63 | 1.25 ± 0.76 | 10 | 0.421 | 0.222 | 0.481 |
| PL6 | 1.05 ± 0.62 | 0.86 ± 0.55 | 10 | 1.29 ± 1.28 | 1.49 ± 1.39 | 10 | 0.151 | 1.000 | 0.166 |
| PL7 | 0.96 ± 0.68 | 0.80 ± 0.61 | 10 | 0.81 ± 0.66 | 0.99 ± 0.80 | 8 | 0.310 | 0.686 | 0.829 |
| PL8 | 1.86 ± 1.23 | 1.54 ± 1.08 | 10 | 0.85 ± 0.49 | 1.00 ± 0.57 | 9 | 0.548 | 0.905 | 0.243 |
| PL9 | 2.40 ± 0.73 | 1.97 ± 0.68 | 9 | 2.23 ± 1.40 | 2.64 ± 1.59 | 9 | 1.000 | 0.413 | 0.340 |
| D1 | 1.79 ± 1.74 | 1.42 ± 1.36 | 10 | 1.04 ± 0.39 | 1.24 ± 0.51 | 10 | 0.032* | 0.222 | 0.529 |
| D2 | 1.06 ± 0.78 | 0.86 ± 0.62 | 10 | 0.63 ± 0.42 | 0.76 ± 0.55 | 10 | 0.841 | 0.691 | 0.481 |
| D4 | 1.21 ± 0.54 | 1.01 ± 0.47 | 9 | 1.39 ± 0.67 | 1.66 ± 0.86 | 10 | 0.905 | 0.421 | 0.113 |
| D5 | 1.73 ± 1.33 | 1.47 ± 1.17 | 10 | 1.14 ± 0.64 | 1.31 ± 0.67 | 9 | 0.841 | 0.730 | 0.968 |
| CD1 | 0.80 ± 0.41 | 0.64 ± 0.32 | 10 | 0.76 ± 0.53 | 0.91 ± 0.65 | 10 | 0.691 | 0.841 | 0.393 |
| CD2 | 0.87 ± 0.51 | 0.72 ± 0.45 | 10 | 0.60 ± 0.43 | 0.73 ± 0.55 | 10 | 0.548 | 0.691 | 0.853 |
| CD3 | 0.59 ± 0.32 | 0.50 ± 0.29 | 10 | 0.78 ± 0.30 | 0.93 ± 0.39 | 10 | 0.548 | 1.000 | 0.019* |
| CD4 | 1.19 ± 0.49 | 1.00 ± 0.49 | 10 | 1.52 ± 0.58 | 1.77 ± 0.64 | 10 | 0.151 | 1.000 | 0.015* |
| CD5 | 0.97 ± 0.73 | 0.80 ± 0.62 | 10 | 0.80 ± 0.50 | 0.92 ± 0.55 | 10 | 0.841 | 0.222 | 0.684 |
| CD6 | 0.80 ± 0.62 | 0.66 ± 0.50 | 9 | 0.53 ± 0.19 | 0.63 ± 0.22 | 10 | 0.064 | 0.841 | 0.661 |
| V1 | 2.29 ± 1.46 | 1.90 ± 1.28 | 10 | 1.68 ± 0.79 | 1.98 ± 0.99 | 10 | 0.421 | 0.841 | 0.579 |
| V2 | 1.51 ± 0.90 | 1.20 ± 0.68 | 10 | 0.82 ± 0.38 | 0.96 ± 0.41 | 10 | 0.008* | 0.095 | 0.579 |
| V3 | 1.69 ± 0.95 | 1.33 ± 0.69 | 10 | 1.12 ± 1.02 | 1.25 ± 1.04 | 10 | 0.691 | 0.691 | 0.796 |
| V4 | 1.38 ± 0.93 | 1.14 ± 0.80 | 10 | 0.62 ± 0.36 | 0.74 ± 0.42 | 10 | 0.421 | 0.691 | 0.315 |
| V5 | 1.15 ± 0.52 | 0.94 ± 0.42 | 10 | 0.88 ± 0.57 | 1.02 ± 0.62 | 10 | 0.691 | 0.691 | 0.971 |
| V6 | 1.04 ± 0.93 | 0.86 ± 0.80 | 10 | 1.23 ± 1.13 | 1.43 ± 1.30 | 10 | 0.691 | 0.222 | 0.143 |
| V7 | 0.74 ± 0.41 | 0.60 ± 0.34 | 10 | 1.10 ± 0.96 | 1.26 ± 1.08 | 9 | 0.548 | 0.556 | 0.400 |
| V8 | 0.94 ± 0.40 | 0.76 ± 0.31 | 9 | 0.96 ± 0.56 | 1.11 ± 0.60 | 10 | 1.000 | 0.691 | 0.182 |
| MD1 | 5.22 ± 1.09 | 4.26 ± 0.94 | 10 | 4.09 ± 0.63 | 4.81 ± 0.81 | 10 | 0.691 | 0.032* | 0.218 |
| MD2 | 1.84 ± 1.03 | 1.52 ± 0.80 | 9 | 1.27 ± 0.64 | 1.48 ± 0.71 | 10 | 0.191 | 1.000 | 0.720 |
| MD3 | 1.33 ± 0.44 | 1.10 ± 0.35 | 8 | 1.44 ± 0.61 | 1.70 ± 0.69 | 10 | 0.114 | 1.000 | 0.016* |
| Total AL Volume | 123.32 ± 12.03 |  | 10 | 85.37 ± 7.65 |  | 10 | 0.548 | 0.548 | <0.001* |

Volume and relative volume are presented as Mean ± SD. Relative volume was used for statistical comparison to control for variation in the absolute volume in individuals, except for comparing total antennal lobe volumes. Asterisks indicate statistical significance at the level of *P* = 0.05.
