# Supplementary figures and images for: An updated antennal lobe atlas for the yellow fever mosquito Aedes aegypti
Source: PLoS Negl Trop Dis. 2020 Oct 20;14(10):e0008729. doi: 10.1371/journal.pntd.0008729 (PMC7575095; doi:10.1371/journal.pntd.0008729)

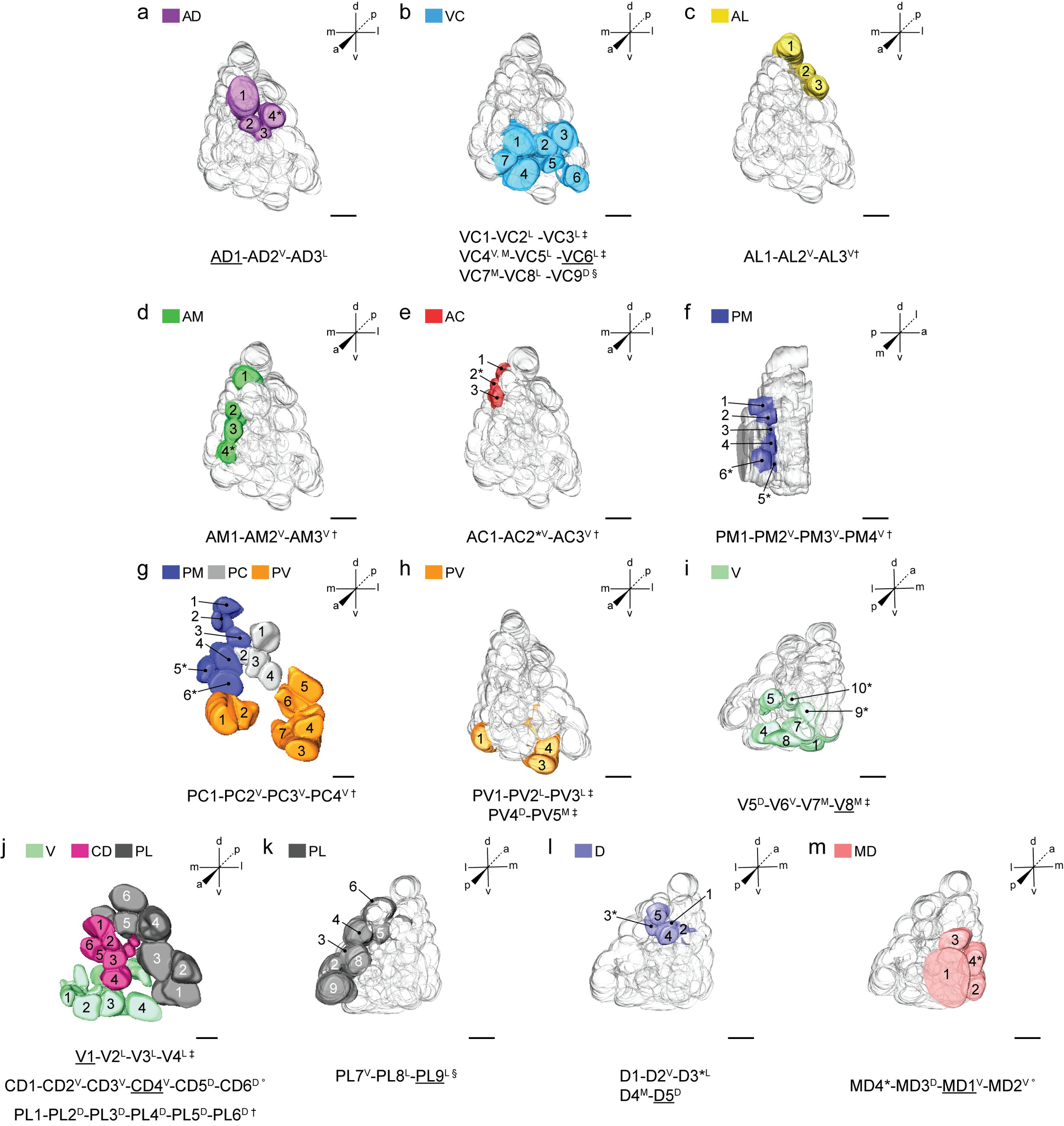

Supplement: S1 Fig — (A–M) Glomeruli within intra-group arrays belonging to the thirteen color-coded spatial groups are highlighted from various perspectives of the antennal lobe. Scale bars = 10 μm. All remaining glomeruli were made transparent to highlight these example arrays and their typical spatial position. Textual annotations that denote the typical spatial arrangement of glomeruli within intra-group glomerular arrays are shown below each perspective of the model lobe. Underlined glomeruli in arrays (AD1, VC6, D5, V1, V8, MD1, CD4 and PL9) are landmarks that facilitate identification of other glomeruli based on their relative spatial position. Glomeruli are typically arrayed in columns†, rows‡, circles°, or randomly arrayed and numbered based on depth§. Each glomerulus may occupy a medialM, lateralL, dorsalD or ventralV position relative to the preceding glomerulus in its array. Landmark glomeruli are underlined. Commonly observed variant glomeruli are indicated by an asterisk*. A full series of intra- and inter-group glomerular arrays are detailed in Table 2. (TIF) [file pntd.0008729.s004.tif]

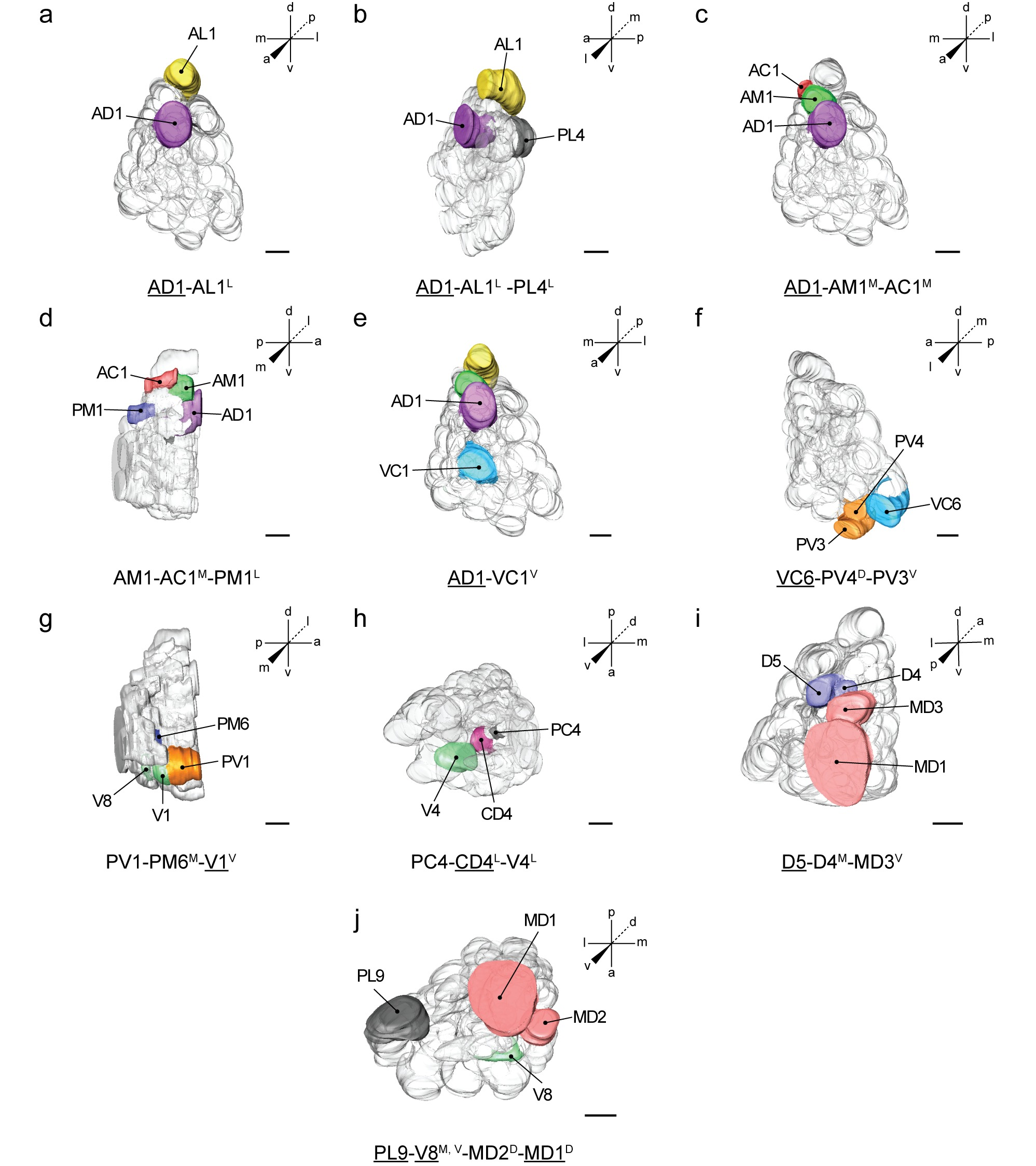

Supplement: S2 Fig — (A–J) Glomeruli within inter-group arrays are highlighted from various perspectives of the antennal lobe and are color-coded on the 3D model according to their spatial group. Scale bars = 10 μm. All remaining glomeruli were made transparent to highlight these example arrays and their typical spatial position. Glomeruli are arranged in layers from the anterior through posterior sections of the antennal lobe. Textual annotations that denote the typical spatial arrangement of glomeruli within an inter-group glomerular array are shown below each perspective of the model lobe. Underlined glomeruli in arrays (AD1, VC6, D5, V1, V8, MD1, CD4 and PL9) are landmarks that facilitate identification of other glomeruli based on their relative spatial position. Each glomerulus may occupy a medialM, lateralL, dorsalD or ventralV position relative to the preceding glomerulus in its array. A full series of intra- and inter-group glomerular arrays are detailed in Table 2. (TIF) [file pntd.0008729.s005.tif]

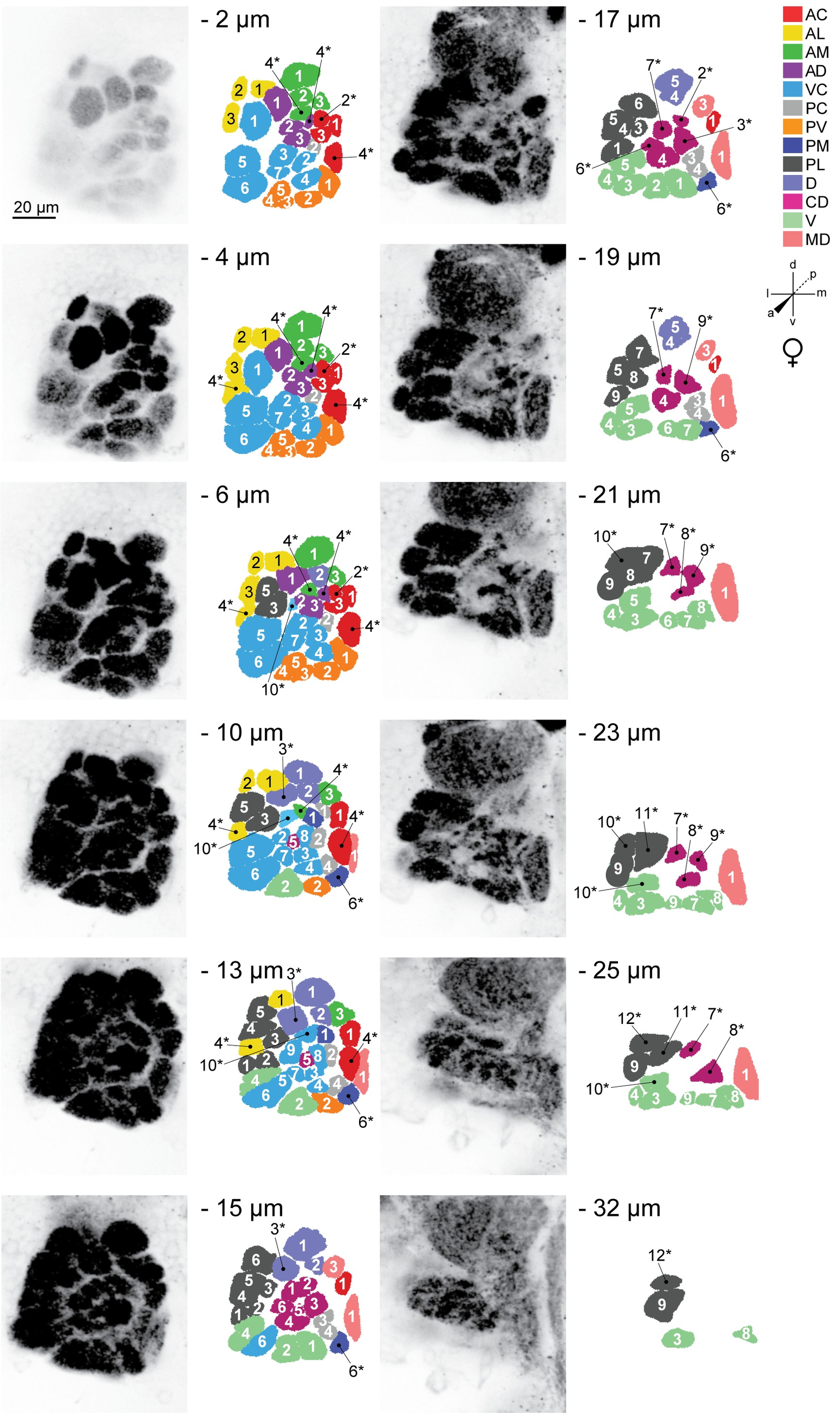

Supplement: S3 Fig — Twelve frontal planes from a total of 35 images taken at 1μm intervals were selected for illustration of the typical geometric arrangement of glomeruli; Scale bar, 20 μm. The depth of each confocal slice is indicated. Glomeruli within each reconstructed slice are color-coded according to their predicted spatial group. Glomeruli are numbered, with 61 out of 63 spatially invariant glomeruli evident in the twelve antennal lobe slices depicted here. Variant glomeruli are indicated by asterisks. (TIF) [file pntd.0008729.s006.tif]

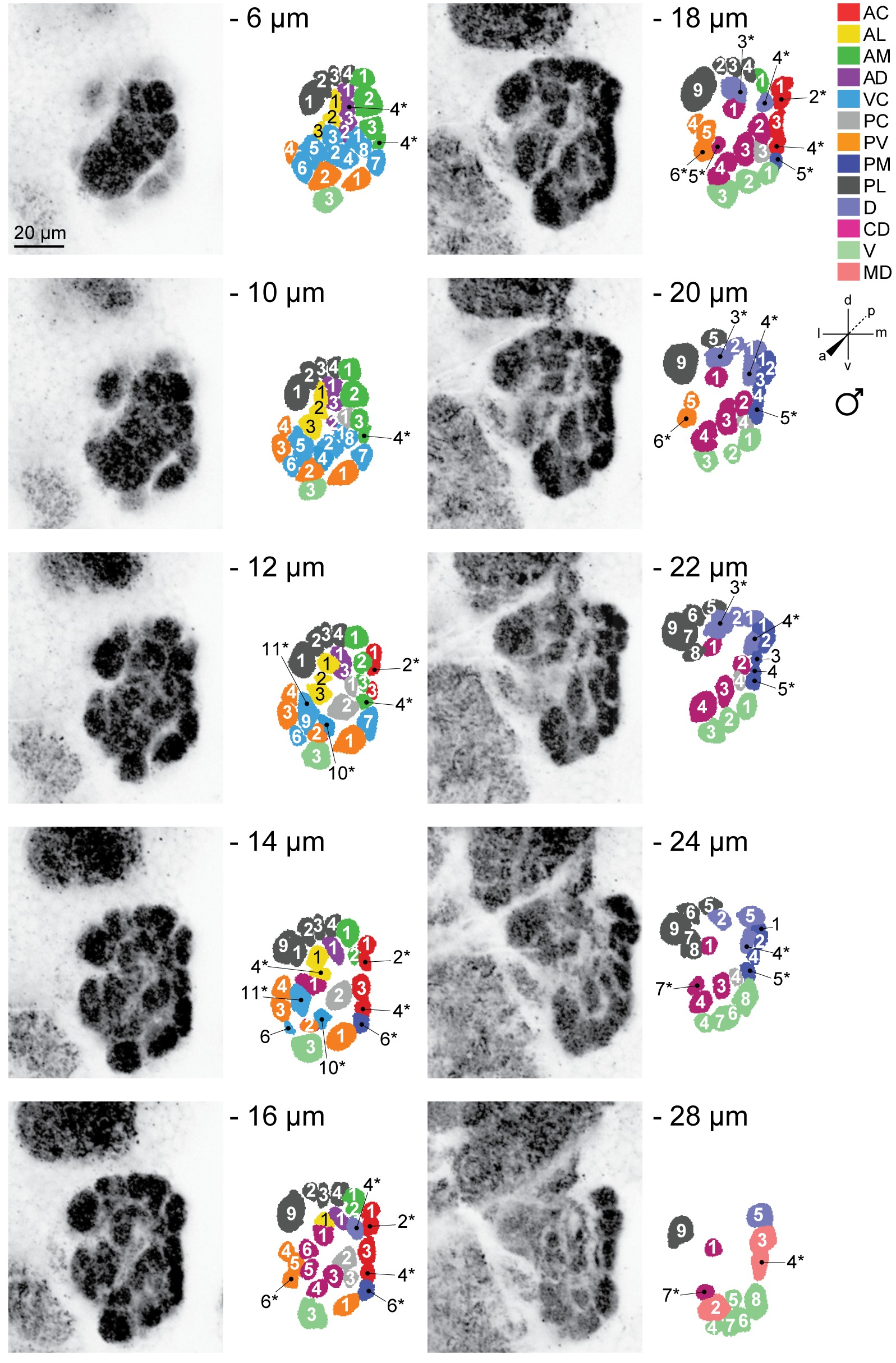

Supplement: S4 Fig — Ten frontal planes from a total of 40 images taken at 1μm intervals were selected for illustration of the typical geometric arrangement of glomeruli; Scale bar, 20 μm. The depth of each confocal slice is indicated. Glomeruli within each reconstructed slice are color-coded according to their predicted spatial group. Glomeruli are numbered, with 63 out of 63 spatially invariant glomeruli evident in the ten antennal lobe slices depicted here. Variant glomeruli are indicated by asterisks. (TIF) [file pntd.0008729.s007.tif]

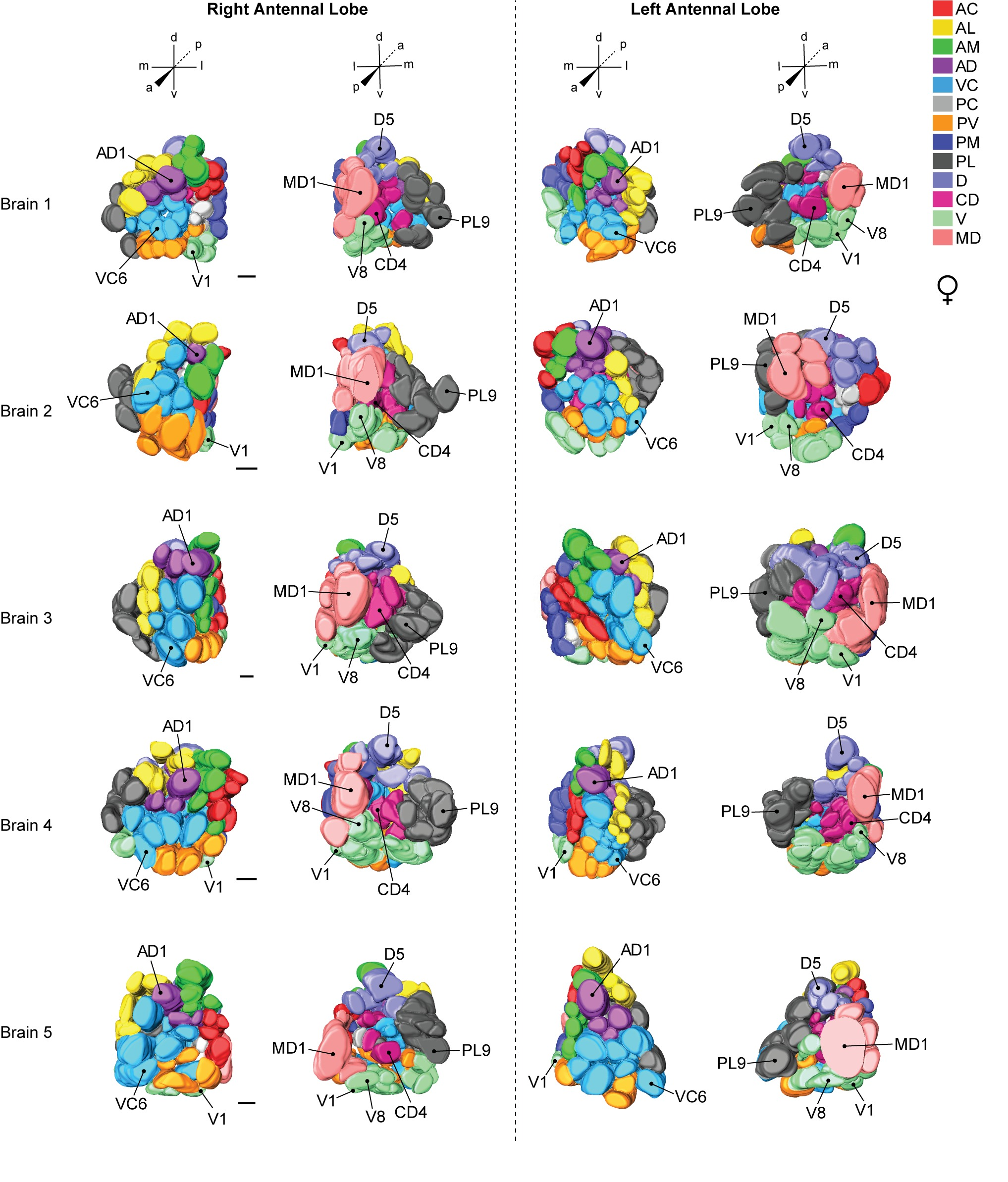

Supplement: S5 Fig — Three dimensional models of the right and left antennal lobes from 5 brain samples of LVPib12 females stained with nc82 antibody are shown. Anterior and posterior perspectives of each antennal lobe reconstruction are illustrated. Glomeruli are color-coded in the 3D models according to their spatial group. Landmark glomeruli are labelled on all reconstructed antennal lobes and include AD1, VC6 located on the anterior surface and D5, V1, V8, MD1, CD4 and PL9 on the posterior surface of the antennal lobe. Scale bars = 10 μm. (TIF) [file pntd.0008729.s008.tif]

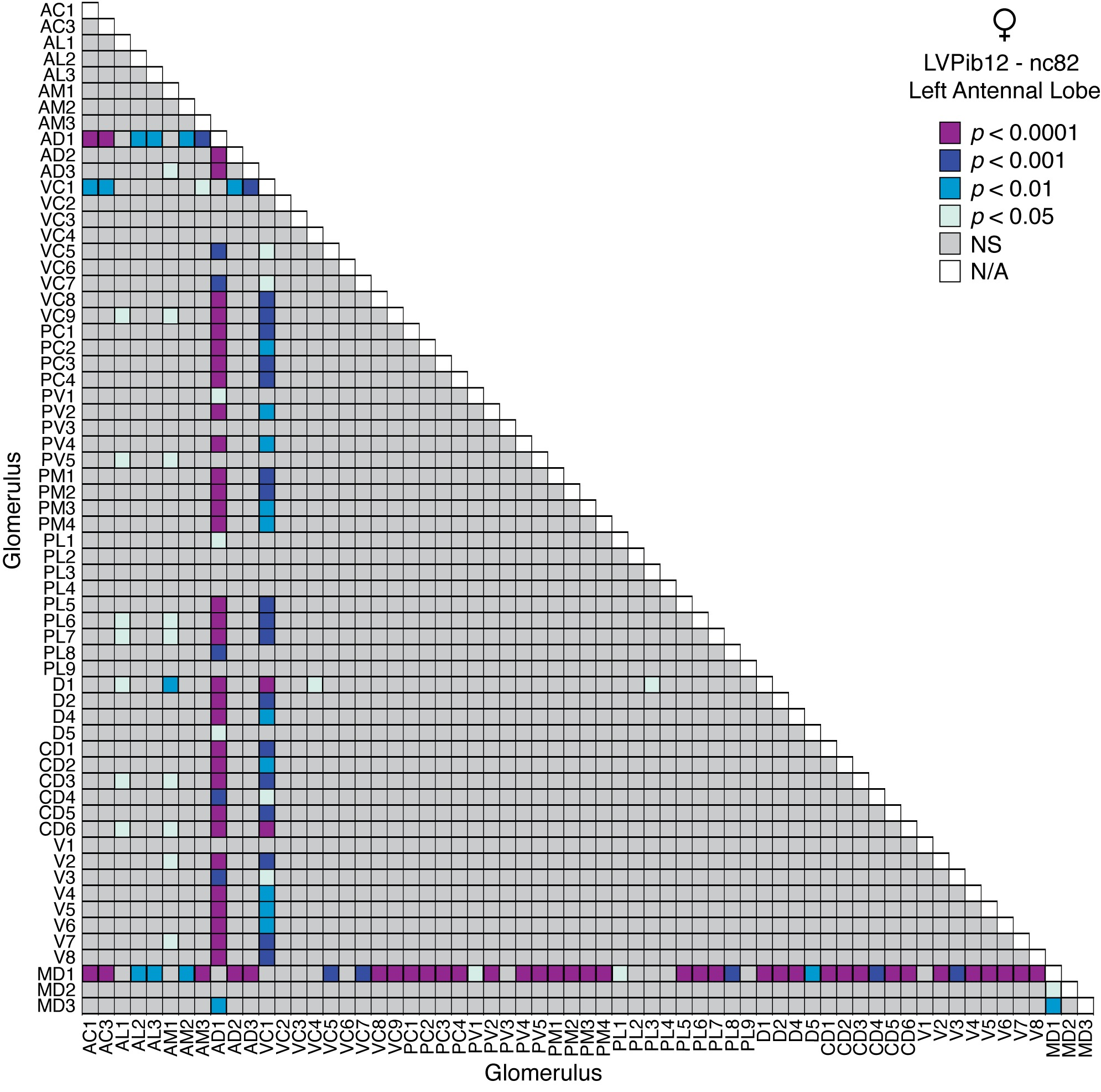

Supplement: S6 Fig — AD1, VC1 and MD1 typically had larger volumes when compared with all other glomeruli. Glomerular means in this lobe differed significantly as determined by one-way ANOVA: F (62, 247) = 5.412, P<0.0001 (n = 5 brains). Abbreviations: NS (not significant). N/A (comparisons between volumetric means of the same glomerulus are not applicable). (TIF) [file pntd.0008729.s009.tif]

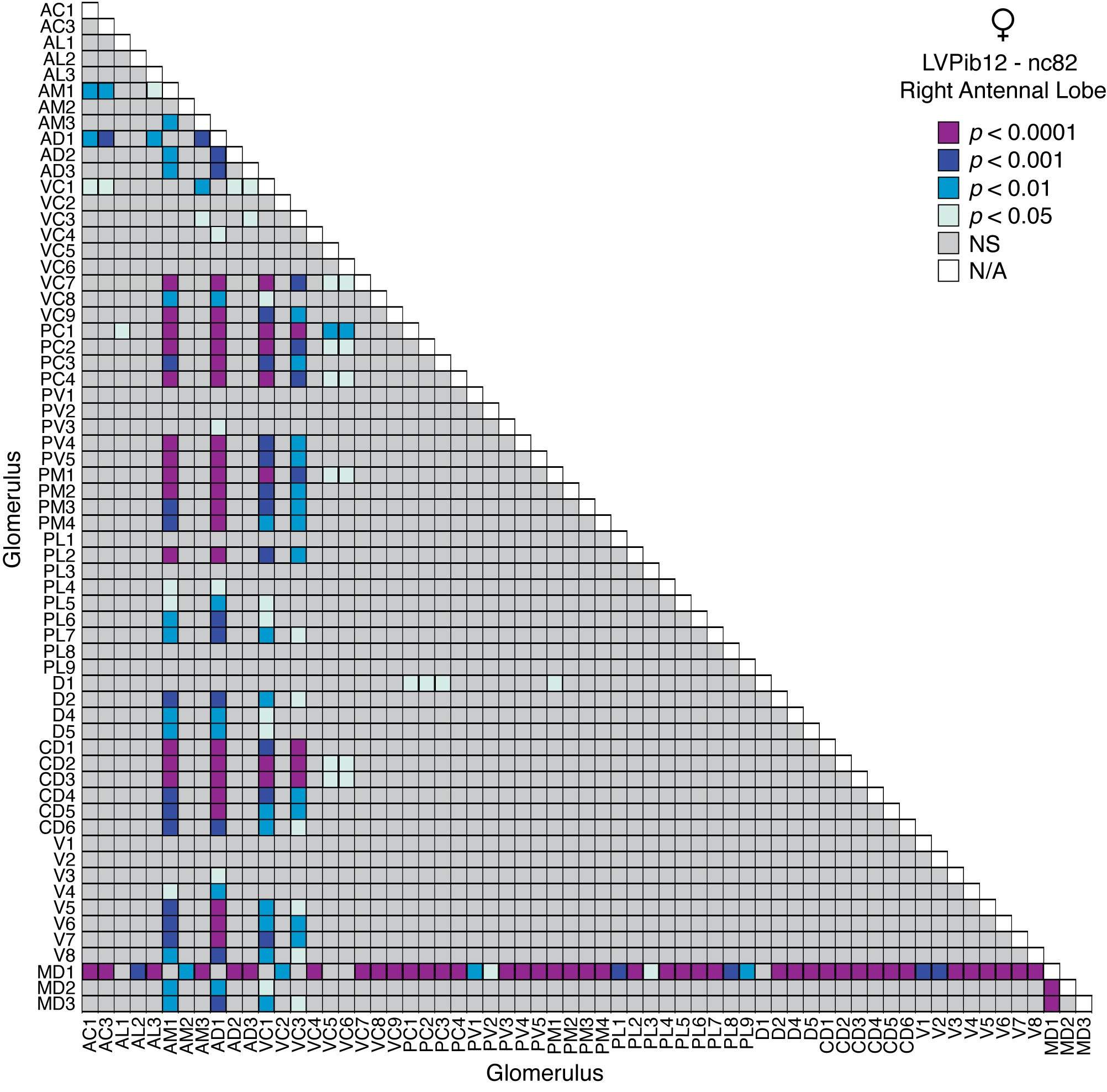

Supplement: S7 Fig — Multiplicity adjusted P values are plotted for each comparison. AM1, AD1, VC1, VC3 and MD1 typically had larger volumes when compared with all other glomeruli. Glomerular means in this lobe differed significantly as determined by one-way ANOVA: F (62, 242) = 6.521, P<0.0001 (n = 5 brains). Abbreviations: NS (not significant). N/A (comparisons between volumetric means of the same glomerulus are not applicable). (TIF) [file pntd.0008729.s010.tif]

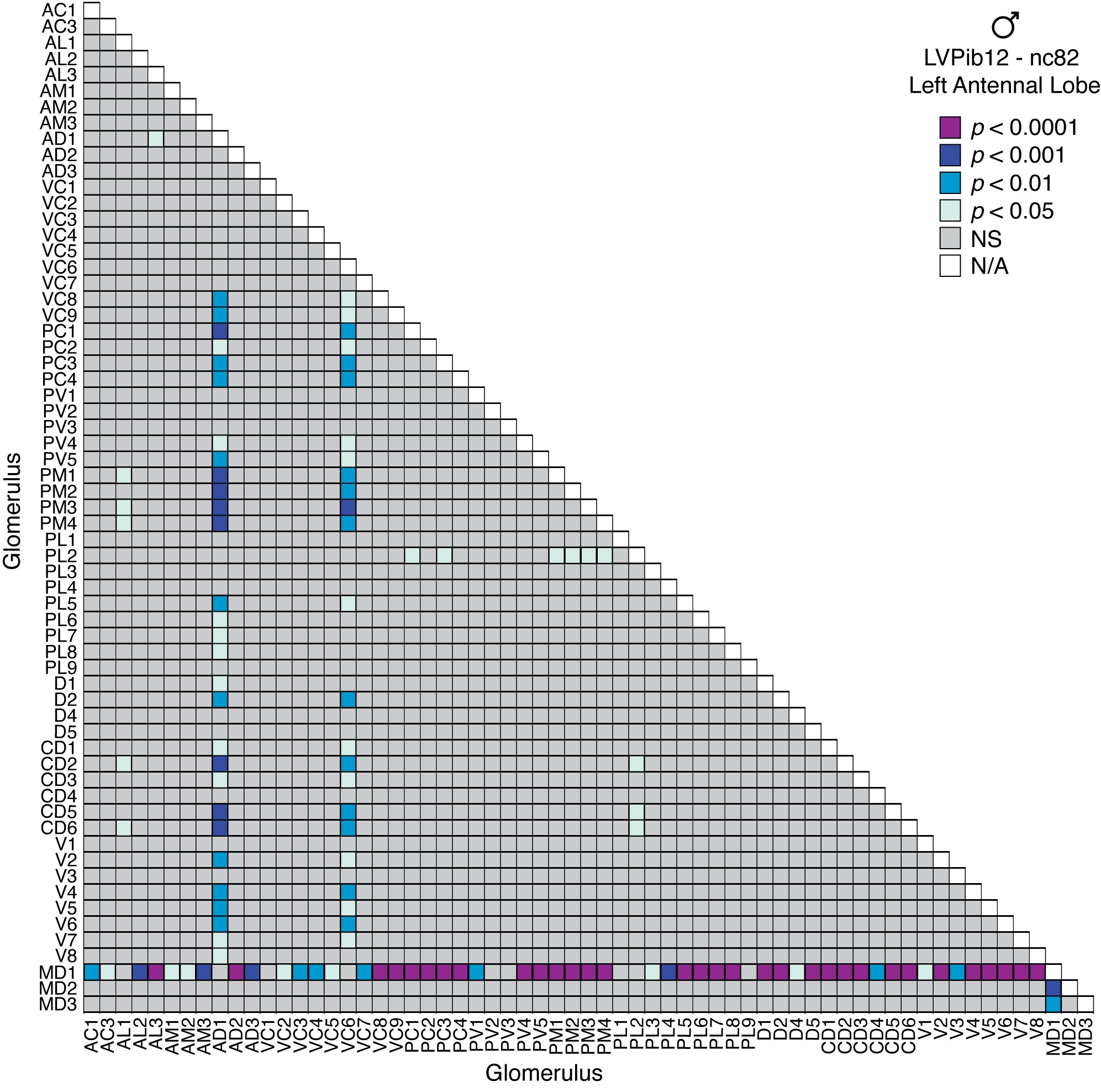

Supplement: S8 Fig — Multiplicity adjusted P values are plotted for each comparison. AD1, VC6 and MD1 typically had larger volumes when compared with all other glomeruli. Glomerular means in this lobe differed significantly as determined by one-way ANOVA: F (62, 241) = 4.472, P<0.0001 (n = 5 brains). Abbreviations: NS (not significant). N/A (comparisons between volumetric means of the same glomerulus are not applicable). (TIF) [file pntd.0008729.s011.tif]

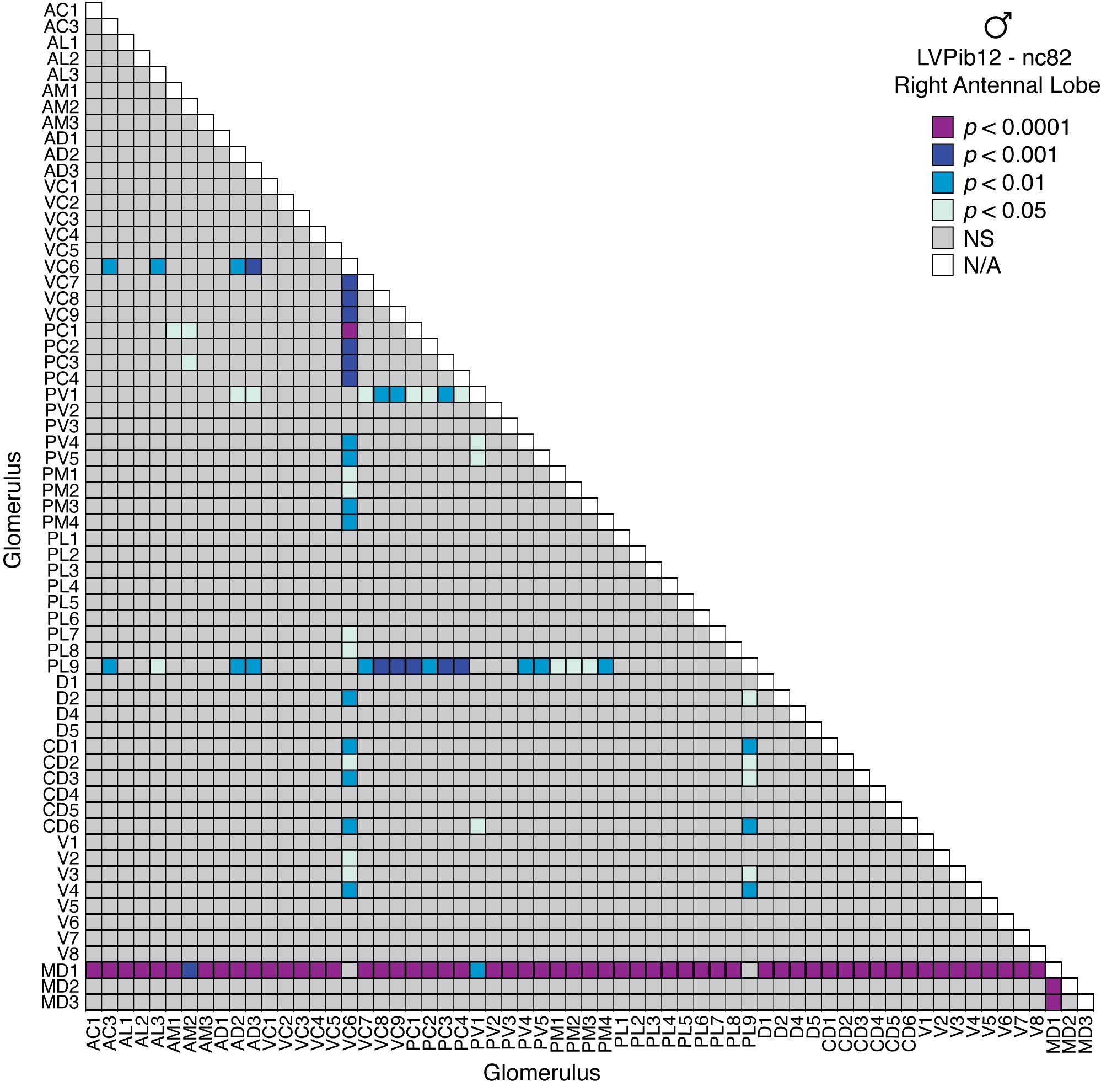

Supplement: S9 Fig — Multiplicity adjusted P values are plotted for each comparison. VC6 and MD1 typically had larger volumes when compared with all other glomeruli. Glomerular means in this lobe differed significantly as determined by one-way ANOVA: F (62, 245) = 5.404, P<0.0001 (n = 5 brains). Abbreviations: NS (not significant). N/A (comparisons between volumetric means of the same glomerulus are not applicable). (TIF) [file pntd.0008729.s012.tif]

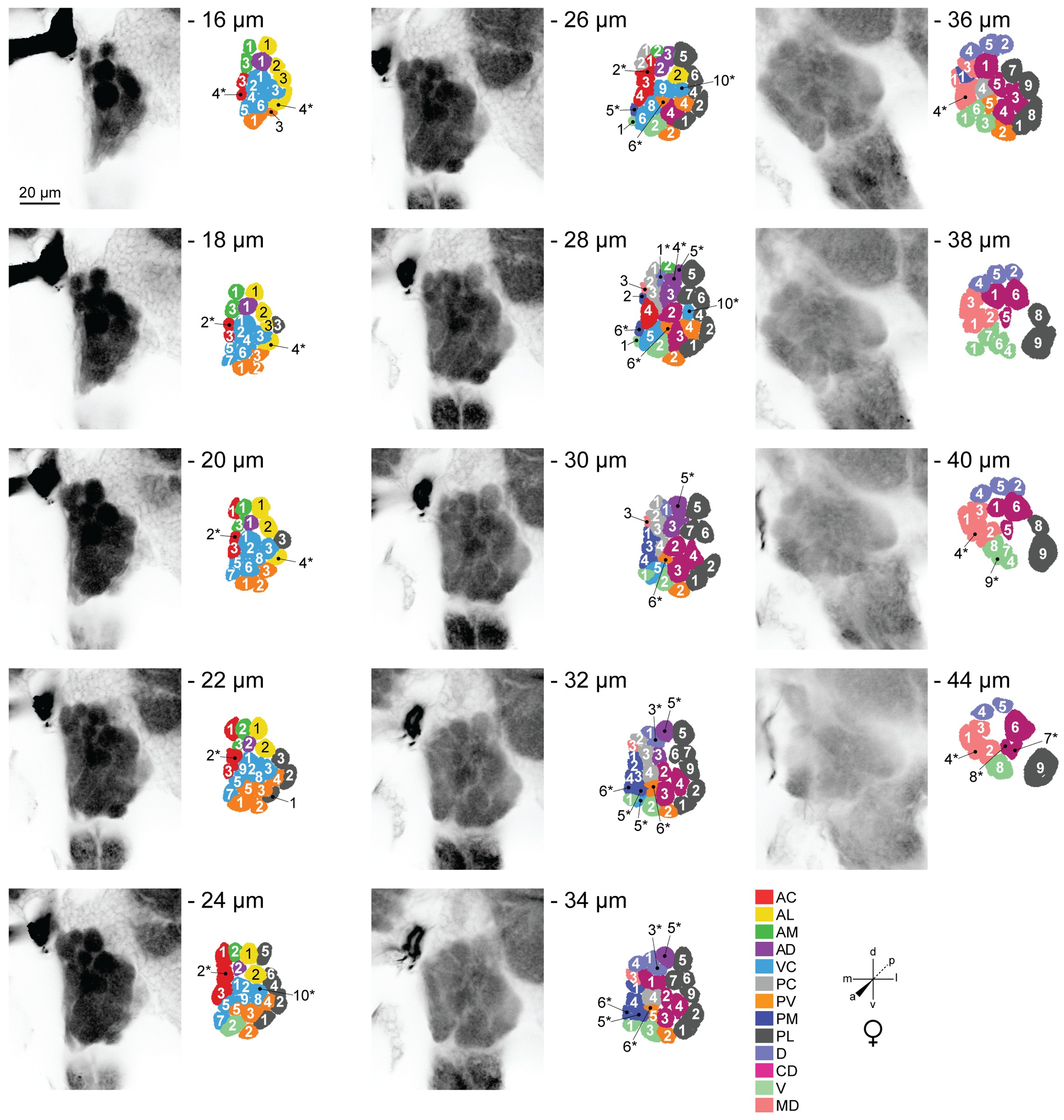

Supplement: S10 Fig — Fourteen frontal planes from a total of 45 images taken at 1μm intervals were selected for illustration of the typical geometric arrangement of glomeruli; Scale bar, 20 μm. The depth of each confocal slice is indicated. Glomeruli within each reconstructed slice are color-coded according to their predicted spatial group. Glomeruli are numbered, with 63 out of the 63 spatially invariant glomeruli evident in the fourteen antennal lobe slices depicted here. Variant glomeruli are indicated by asterisks. (TIF) [file pntd.0008729.s013.tif]

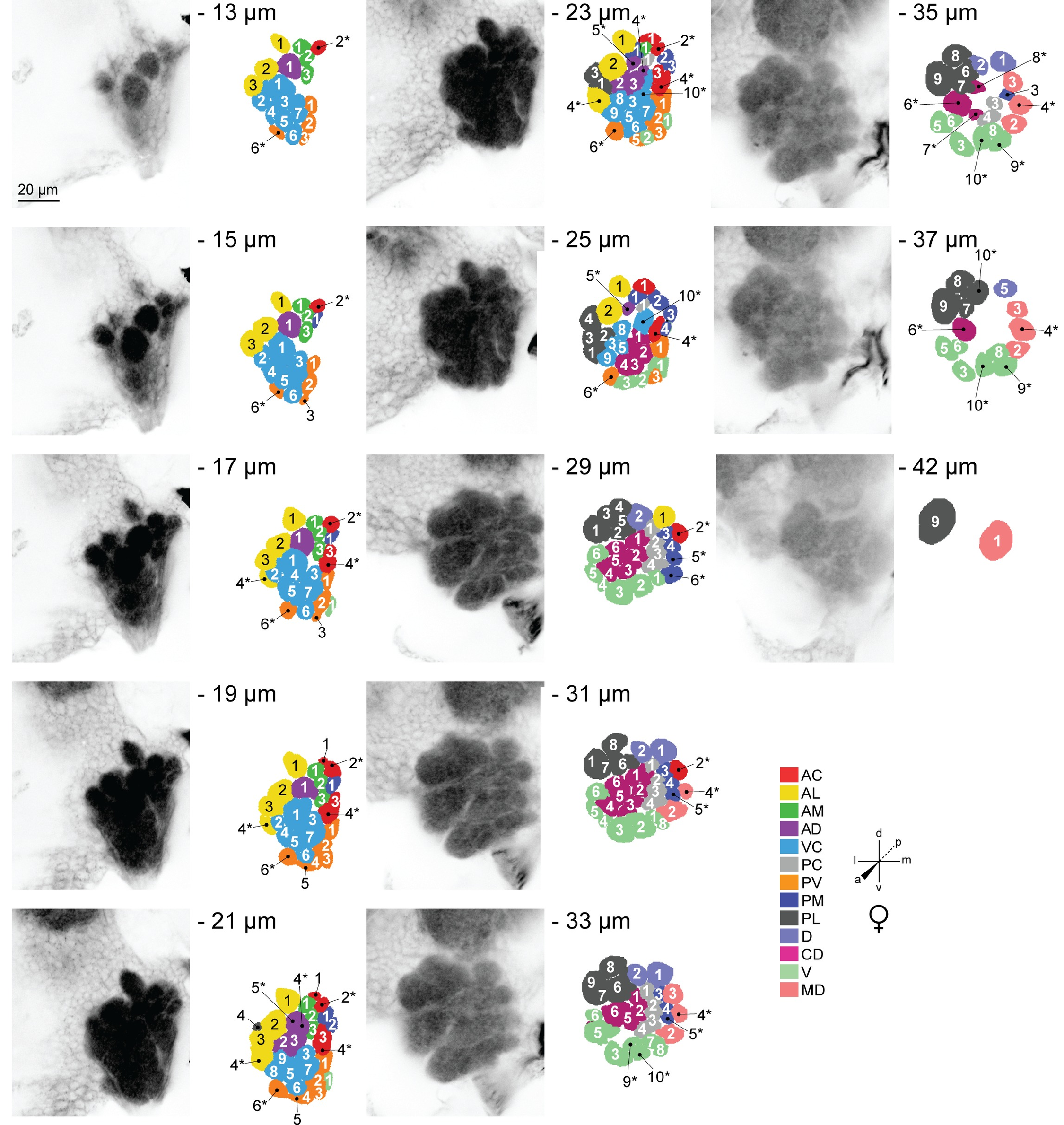

Supplement: S11 Fig — Thirteen frontal planes from a total of 45 images taken at 1μm intervals were selected for illustration of the typical geometric arrangement of glomeruli; Scale bar, 20 μm. The depth of each confocal slice is indicated. Glomeruli within each reconstructed slice are color-coded according to their predicted spatial group. Glomeruli are numbered, with 63 out of the 63 spatially invariant glomeruli evident in the thirteen antennal lobe slices depicted here. Variant glomeruli are indicated by asterisks. (TIF) [file pntd.0008729.s014.tif]
